# Exposure to endosulfan influences sperm competition in *Drosophila melanogaster*

**DOI:** 10.1038/srep07433

**Published:** 2014-12-11

**Authors:** Snigdha Misra, Ajay Kumar, Ch. Ratnasekhar, Vandana Sharma, Mohana Krishna Reddy Mudiam, Kristipati Ravi Ram

**Affiliations:** 1 Embryotoxicology, CSIR-Indian Institute of Toxicology Research, M.G. Marg, Lucknow 226001, Uttar Pradesh, India; 2 Analytical Chemistry, CSIR-Indian Institute of Toxicology Research, M.G. Marg, Lucknow 226001, Uttar Pradesh, India; 3 Academy of Scientific and Innovative Research (AcSIR), CSIR-IITR campus, Lucknow 226001, UP, India

## Abstract

Dwindling male fertility due to xenobiotics is of global concern. Accordingly, male reproductive toxicity assessment of xenobiotics through semen quality analysis in exposed males, and examining progeny production of their mates is critical. These assays, in part, are biased towards monogamy. Females soliciting multiple male partners (polyandry) is the norm in many species. Polyandry incites sperm competition and allows females to bias sperm use. However, consequences of xenobiotic exposure to the sperm in the light of sperm competition remain to be understood. Therefore, we exposed *Drosophila melanogaster* males to endosulfan, and evaluated their progeny production as well as the ability of their sperm to counter rival control sperm in the storage organs of females sequentially mated to control/exposed males. Endosulfan (2 μg/ml) had no significant effect on progeny production and on the expression of certain genes associated with reproduction. However, exposed males performed worse in sperm competition, both as 1^st^ and 2^nd^ male competitors. These findings indicate that simple non-competitive measures of reproductive ability may fail to demonstrate the harmful effects of low-level exposure to xenobiotics on reproduction and advocate consideration of sperm competition, as a parameter, in the reproductive toxicity assessment of xenobiotics to mimic situations prevailing in the nature.

Indiscriminate use of thousands of synthetic chemicals and their inadvertent release to the environment led to tremendous modification in the abiotic chemistry of the environment. Several reports in the past 20–30 years reflect the hazardous impact of xenobiotics on male fertility^1^ and the deterioration of male reproductive health at an exponential rate with every passing decade^2^,^3^,^4^. Moreover, this trend due to adult/developmental exposure to xenobiotics is not limited to higher organisms (including human) but is observed in males from all taxonomic ranks across the animal kingdom. Consequently, the reproductive toxicity assessment of various environmental agents has led to evaluation of their potential to hamper male reproductive health^5^. The adverse effects of various environmental agents on the male fertility are well documented through the standard semen quality analysis (including sperm counts, sperm morphology and motility) in males exposed to chemicals and/or by assessing progeny production of their mates^6^. The semen quality assessment does not account for the sperm fate in the female environment and the assay of progeny production is biased towards monogamy as the design of the assay involves pairing of one male with a female^7^. However, in nature, females of most animal species actively soliciting copulations with multiple males^8^,^9^ to optimize the offspring number^10^ is quite common. Although the adaptive significance of the same to females is unclear, polyandry is a strategy suggested to reduce the risk of population from being extinct^11^. Despite this, there is paucity of knowledge on the adverse reproductive consequences of exposure to xenobiotics in the context of polyandry.

Polyandry incites post-copulatory male-male competition inside the female reproductive tract in the form of sperm competition^12^ with sperm from two or more males trying to fertilize the ovum, to produce genetically variable offspring in stochastic environmental conditions. The success in sperm competition depends upon the number of sperm displaced and the quality of the ejaculate provided by the male^13^,^14^. Therefore, in the present study, using *Drosophila melanogaster* as a model organism, we evaluated the consequence of the environmental chemical exposure on sperm competition. *Drosophila melanogaster*, with its unique suite of genetic tools, has proven to be an excellent model for toxicological studies in general^15^,^16^ and male reproductive toxicity assessment in particular^17^,^18^,^19^. Further, the use of Drosophila as a model is in line with the recommendations of European Centre for Validation of Alternate Methods (ECVAM) for the use of lower vertebrates and invertebrates as alternatives to existent animal models^20^. In addition, *Drosophila melanogaster* transgenic lines harboring enhanced green fluorescent protein (EGFP) or *Discosoma* red fluorescent protein (dsRed) labeled sperm permit unambiguous differentiation of sperm from competing rivals, within the mated females' reproductive tract to analyze sperm precedence, displacement of pre-existing sperm by second males, and biased use of competing sperm coming from rival mates, in the process of fertilization^21^.

In this study, we analyzed the impact of exposure to endosulfan on sperm competition. Endosulfan is a broad spectrum non-systemic persistent organic pollutant (POP), which binds to sediments of the aquatic/terrestrial matrices and tends to bioaccumulate in the tissues of the organisms inhabiting the contaminated matrix. Endosulfan is known to hamper male fertility^22^. The concentrations of endosulfan reported vary in different matrices of environment from 1.7 μg/ml in water bodies, 0.3–34.86 μg/ml in soil to 0.36–212.28 μg/ml in fruits^23^. Developmental exposure of Drosophila to endosulfan was shown to induce ROS generation, oxidative stress and xenobiotic metabolism markers^24^. Here, by exposing transgenic Drosophila adult males to environmentally relevant concentration(s) of endosulfan, we show that exposure to endosulfan indeed affects sperm competition. In both defensive and offensive sperm competition, sperm from exposed males failed to perform at control levels as witnessed by their proportions in the sperm storage organs. This was further validated, at the organismal level, and we observed that the exposed males, sired fewer progeny (P_1_/P_2_), irrespective of they being the first or the second mate, when compared to their control mates. Alarmingly, endosulfan, at this concentration and exposure duration, has no detectable effect on the number of progeny (produced by the exposed male when mated to control females), sperm storage and expression of genes encoding male reproductive proteins in Drosophila. These findings suggest the vulnerability of sperm competition over other reproductive components to xenobiotic exposure. Moreover, the study indicates that simple non-competitive measures of reproductive ability may fail to demonstrate the harmful effects of environmental agents on reproduction and highlights the importance of competitive measures of reproductive ability to account for the scenario prevailing in the nature.

## Results

### Males exposed to endosulfan perform poorly in defensive sperm competition when compared to control males

**Figure 1:**
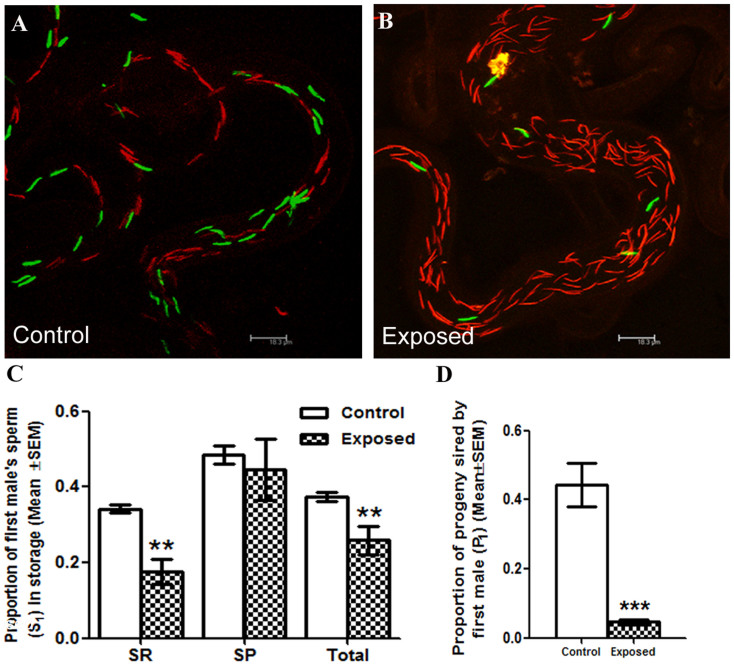
Assessment of the ability of sperm from males exposed to endosulfan (EGFP) to defend against the rival control sperm. Panel A depicts EGFP (green) and dsRed (red) sperm at 2 h ASSM in the seminal receptacle of *w*^1118^ females first mated to control Prot B-EGFP males and remated to control Prot B-dsRed males, as visualized under confocal microscope. Panel B represents sperm from different males in the seminal receptacles of females first mated to Prot B-EGFP males exposed to 2 μg/ml endosulfan and subsequently mated to control Prot B-dsRed males. Panel C gives the proportion of first male sperm (S_1_) in different sperm storage organs, namely seminal receptacle (SR) and spermathecae (SP) and among sum of sperm (represented by Total **p < 0.01; N = 15–20) stored in seminal receptacle, and spermathecae. Panel D represents the proportion of progeny sired by ProtamineB-EGFP males when they are the first to mate (P_1_) under control as well as exposure conditions (2 μg/ml) with *w*^1118^ males as the second rival mates. P1 was calculated as the proportion of red eyed progeny (sired by Prot B-EGFP males) over the total of red and white eyed (sired by *w*^1118^) progeny produced by the female/10days ASSM (***p < 0.001; N = 25–30).

To evaluate the ability of sperm from males exposed to endosulfan to defend against the rival control sperm, we mated the *w*^1118^ females to control or exposed EGFP males, and after 3 days, remated these females with dsRed males. Subsequently, we determined the proportion of the first male's (EGFP) sperm (S_1_) to the total sperm (EGFP + dsRed) residing in the storage at 2 h ASSM (After the start of second mating) by counting the sperm in seminal receptacle and paired spermathecae, the two major sperm storage organs in Drosophila. When compared to the S_1_ under control conditions in the seminal receptacle (0.3421 ± 0.01198; Figs. 1A & 1C), we observed significant reduction in the proportion of first male's sperm (S_1_) under exposure conditions (0.1755 ± 0.03226; p = 0.0013; Figs. 1B &1C). In contrast, we observed similar S_1_ levels in spermathecae of females mated to control (0.5017 ± 0.02002) or exposed (0.4454 ± 0.08036; p = 0.9497; Fig. 1C) EGFP males. Yet, the proportion of first male sperm among total sperm in storage (counts including both storage organs) in females mated to EGFP males exposed to endosulfan (0.2592 ± 0.03761; p = 0.0047; Fig. 1C), differed significantly from that in the control group (0.3783 ± 0.01249; Fig. 1C). A similar trend was witnessed in the ratio of EGFP to rival dsRed sperm in the seminal receptacle (p < 0.001; Fig. S1), spermathecae (p = 0.4891; Fig. S1) and among total sperm in storage (p = 0.0025; Fig. S1).

At the organismal level, the proportion of progeny sired by the first male (P_1_) exposed to endosulfan was significantly lower when compared to that of controls (p < 0.001; Fig. 1D) indicating the differential sperm use by the female for the process of fertilization.

### Males exposed to endosulfan sired significantly reduced offspring in offensive sperm competition when compared to control males

**Figure 2:**
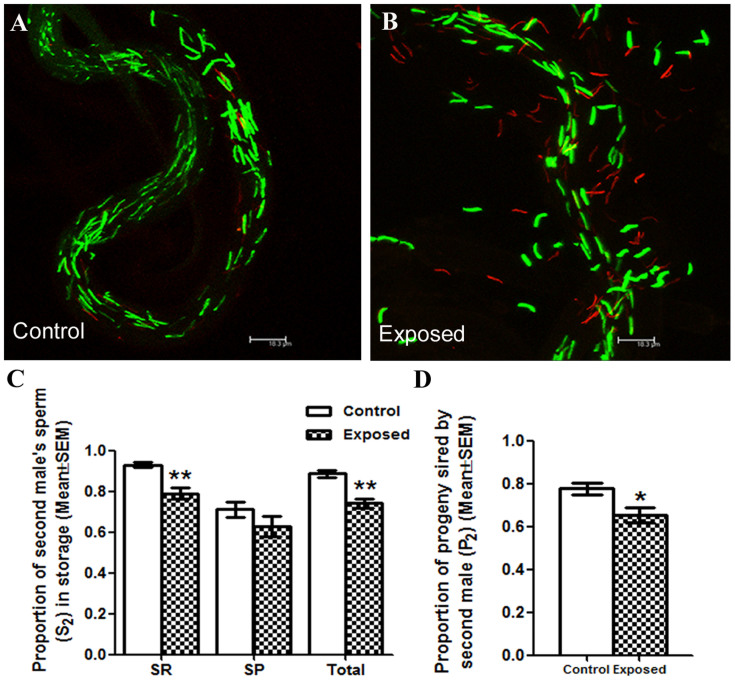
Assessment of the ability of sperm from males exposed to endosulfan (EGFP) to displace the rival control sperm. GFP (green) and dsRed (red) sperm observed at 2 h ASSM in the seminal receptacle of *w*^1118^ females first mated to control Prot B-dsRed males and remated to control Prot B-EGFP males (Panel A) or Prot B-EGFP males exposed to 2 μg/ml endosulfan (Panel B) are represented. Panel C represents the proportion of second male sperm (S_2_) in different sperm storage organs, seminal receptacle (SR), spermathecae (SP) and among the total sperm in storage (**p < 0.01; N = 10–15). Panel D represents the proportion of red eyed progeny sired by Prot B- EGFP (control/exposed) when they were second to mate (P_2_) with females first mated to *w*^1118^ males, out of the total (red + white eyed) progeny/female/10 days ASSM (*p = 0.0245; N = 25–30).

To determine the efficiency of the sperm from males exposed to endosulfan to rival the control sperm from the first mate, we presented control dsRed males to the *w*^1118^ virgin females, as their first mates, and control/exposed EGFP males, as their second mates. Subsequently, we determined the proportion of second male's sperm (S_2_) by counting EGFP and dsRed sperm in the storage organs of these females. Under control conditions, S_2_ in the seminal receptacles was 0.9302 ± 0.014 (Figs. 2A & 2C). However, we observed a significant reduction in the S_2_ (0.7906 ± 0.0278; p = 0.0012; Figs. 2B & 2C) in seminal receptacles of females mated to exposed EGFP males, as their second mates, in comparison to those in controls (see above). On the contrary, similar to our observations in defensive sperm competition, S_2_ in the spermathecae of females mated to control EGFP males (0.7110 ± 0.03814; Fig. 2C) did not differ from that of females to exposed EGFP males, (0.6278 ± 0.04926; p = 0.2343; Fig. 2C). Nevertheless, the difference in the S_2_ levels among total sperm stored by females mated to exposed males (0.7410 ± 0.02408; p = 0.0012; Fig. 2C) was significantly different from that in their controls (0.8868 ± 0.01605; Fig. 2C). In addition, the ratio of EGFP to dsRed in seminal receptacle (p = 0.0054; Fig. S2), spermathecae (p = 0.3922; Fig. S2) and total sperm (p = 0.0015; Fig. S2) is concordant with the observed S_2_ levels. The altered offense capability was also reflected at the organismal level in the proportion of progeny sired by the second male (P_2_) of endosulfan exposure group. When the EGFP males were the second to mate, the proportions of red eyed progeny sired by them under exposed conditions were significantly lower (p = 0.0245; Fig. 2D) when compared to those under control conditions.

### Sperm from males exposed to endosulfan are stored at levels similar to controls in mated females

**Figure 3:**
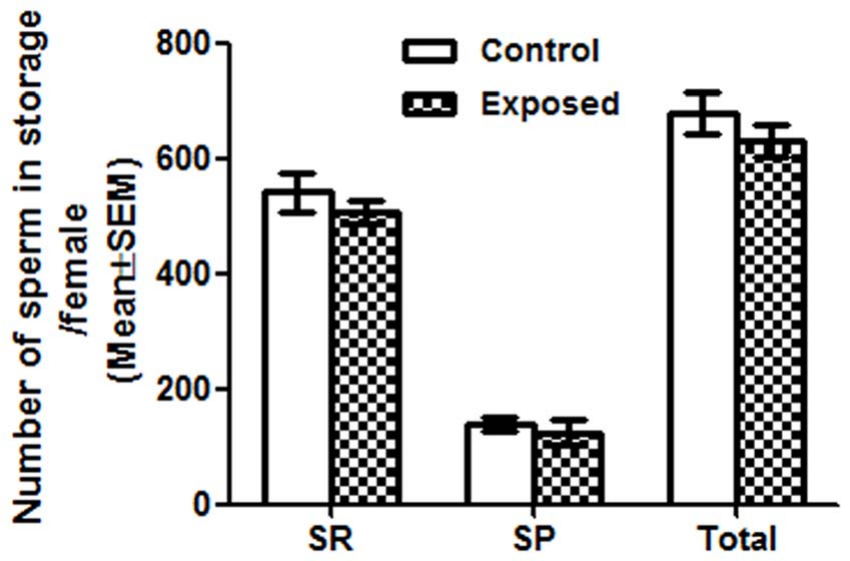
Females store sperm from males exposed to endosulfan at levels similar to controls. We observed similar numbers of sperm in seminal receptacle (SR, p = 0.3917; N = 20–25), spermathecae (SP, p = 0.5534; N = 20–25) in females mated to control or exposed males at 2 h ASM. The total sperm in storage of females (sum of sperm stored in SR, and SP represented by Total) mated to control or exposed males also did not differ from each other.

To determine if the observed effect on sperm competition is a consequence of reduced receipt of sperm, in response to chemical exposure, we quantified the storage levels of sperm in the spermathecae and seminal receptacles of females mated to control or exposed Prot B-EGFP males. We observed that the number of sperm stored in each storage organ and the total number of sperm stored in females mated to control males were comparable to those mated to exposed Prot B-EGFP males (p > 0.05; Fig. 3).

### Endosulfan affects sperm competition at concentrations that do not affect progeny production and the transcripts of certain genes encoding reproductive proteins

**Figure 4:**
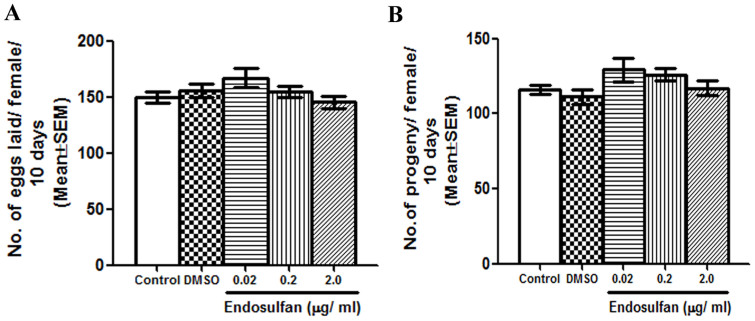
Evaluation of effects of endosulfan on male fertility. We evaluated effects of endosulfan exposure on male fertility by analyzing the number of eggs laid (Panel A; p > 0.05; N = 15–20) and number of progeny (Panel B; p > 0.05; N = 15–20) produced by the females mated to males exposed to different concentrations of endosulfan. At all concentrations, we observed that both these reproductive parameters were comparable among control, solvent control (DMSO) and exposed groups.

**Figure 5:**
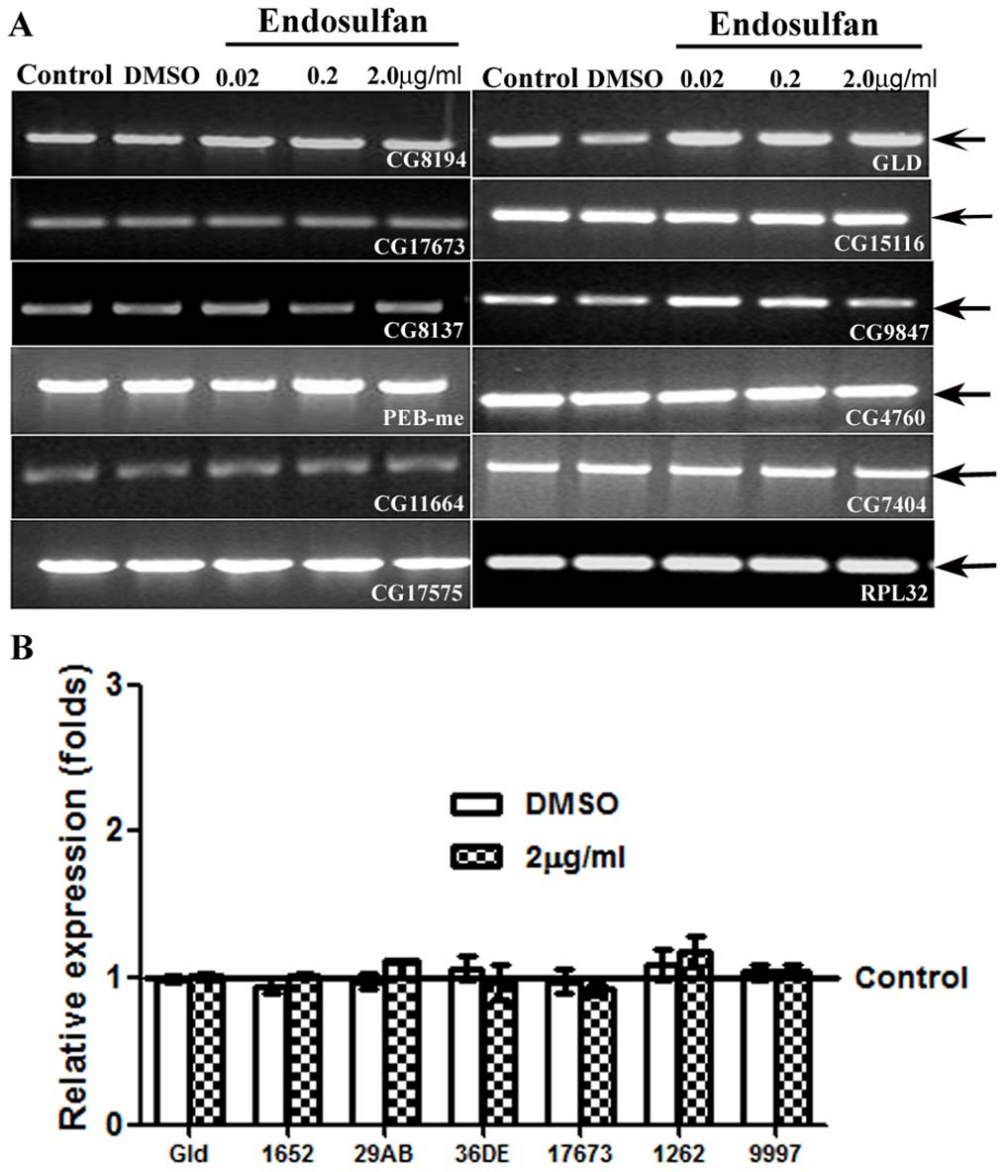
Determination of transcript levels of candidate genes encoding reproductive proteins in Drosophila males exposed to endosulfan and their controls. Panel A depicts the PCR amplicons from semi-quantitative PCR reflecting the transcript levels of candidate genes encoding seminal proteins and/or reproductive proteins in males exposed to 0.02–2 μg/ml of endosulfan and the observed levels were comparable to those in control males. We used RPL32 (RPL 32 panel; Panel A) as an internal control for the quality as well as quantity of the template. The semi-quantitative PCR data were validated by quantifying the expression levels of, GLD and CG17673 through qPCR (Panel B, p > 0.05) in males exposed to 2 μg/ml of endosulfan and their controls as well as vehicular controls (DMSO). The quantities of transcripts of additional genes implicated in sperm competition in Drosophila (CG1262, CG1652, CG9997, Acp36DE, and Acp29AB; Panel B) were also comparable between control and exposed male samples (p > 0.05).

To determine if sperm from the exposed males are viable and are used by their mates, we analyzed the egg production and progeny production of females mated to control or exposed males. We observed that the females mated to males exposed to 0.02 μg/ml (Fig. 4; p > 0.05), 0.2 μg/ml (Fig. 4; p > 0.05), and 2 μg/ml endosulfan (Fig. 4; p > 0.05) laid eggs at levels similar to their controls (Fig. 4A, control and DMSO bars). Similarly, the number of progeny produced by females also did not vary between females mated to males exposed to various concentrations of endosulfan (Fig. 4B; p > 0.05) or their controls (Fig. 4B, control and DMSO bars). In addition, at all concentrations, we observed that transcript levels of sub-set of genes encoding seminal proteins (CG8194, CG17673, CG8137, PEB-me, CG11664, CG17575, GLD, CG15116, CG9847^25^,^26^) or critical for spermatogenesis (CG4760^27^) or expressed in the male reproductive tract (CG7404^28^) were comparable to those in controls (Fig. 5A).

To validate the expression pattern of the candidate genes observed in semi-quantitative transcript analysis, we analyzed the transcript levels of couple of these genes (CG17673, and GLD) through quantitative real time PCR (qPCR). We observed that the expression levels of CG17673, and GLD were similar between controls and exposed males (p > 0.05; Fig. 5B) and supported our semi-quantitative transcript data. Additionally, expression of genes implicated in sperm competition (CG1652, CG9997^29^, CG1262^30^, Acp36DE^31^, and Acp29AB^32^) was also analyzed through qPCR (Fig. 5B). We observed that the transcript levels of all these genes in males exposed to 2 μg/ml of endosulfan were comparable to those in vehicular controls (DMSO) or controls (p > 0.05; Fig. 5B).

### Isomers of endosulfan are detectable only in exposed males but not in their mates

**Figure 6:**
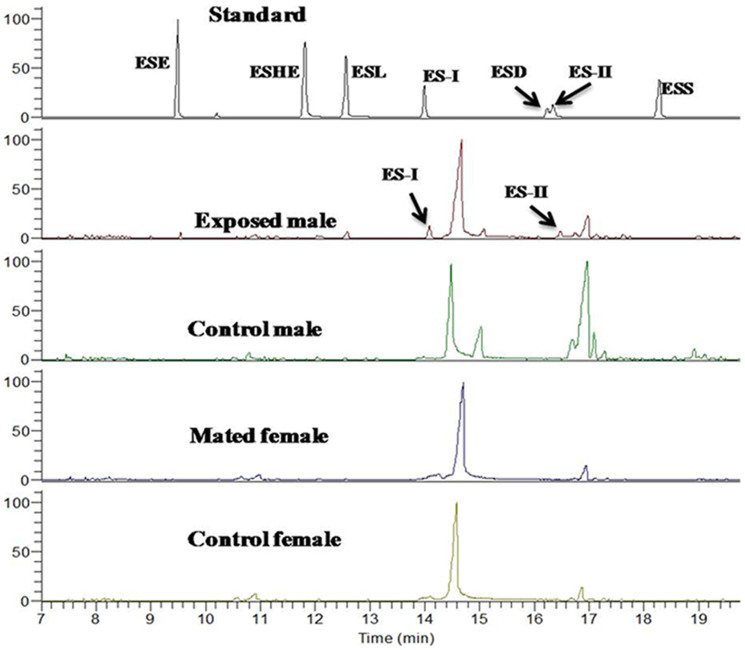
GC-MS chromatogram(s) of males exposed to 2 μg/ml of endosulfan, control males, females mated to exposed male, and control female. Endosulfan (I and II isoforms, marked with black arrows) was detected only in exposed males. ESE (endosulfan ether), ESHE (endosulfan hydroxyl ether), ESL (endosulfan lactone), ES-I (α-endosulfan), ESD (endosulfan diol), and ESS (endosulfan sulphate) were run as standards for determination of RT, peak area and subsequent quantification.

To determine the internalization of the test chemical, we estimated the levels of endosulfan through Gas chromatography coupled with mass-spectrometry (GC-MS/MS) in exposed males and their mates. Two isoforms of endosulfan (ESI, and ESII with reference to peaks obtained in standards; Fig. 6, exposed males panel) were detected only in exposed males. Females mated to exposed males, did not have peaks corresponding to the isoforms (Fig. 6, mated female panel). Similarly we did not observe any peaks corresponding to endosulfan in control males (Fig. 6, control male panel) or their mates (Fig. 6, Control female panel).

## Discussion

The present study was aimed at understanding the consequence of exposure to xenobiotics on the male reproductive performance under competitive conditions. The rationale for the assessment of the toxicity potential of xenobiotics to hamper male fertility under competitive conditions stems from the fact that reproductive success of males in majority of the species under natural conditions is determined by their ability to rival the competing males either at the organismal or at the sub-organismal level. Despite this, the effect of exposure to xenobiotics on this phenomenon remains neglected. Therefore, in the present study, using *Drosophila melanogaster* as a model, we evaluated the effect of a xenobiotic, endosulfan, on male-male competition at organismal/sub-organismal level by examining sperm competition, which is an important component of male reproductive success^33^,^34^,^35^.

The potential of one's sperm pool to defend against and/or displace the sperm from rival pool during sperm competition determines the reproductive fitness of the male. Significantly reduced S_1_ of males exposed to endosulfan when compared with that of controls in the present study, suggests the adverse effects of endosulfan on the ability of sperm to defend against the rival sperm. Similarly, the significantly reduced S_2_ reflects the adverse effect of endosulfan on sperm offence. Interestingly, in both cases, competition was evident among the sperm stored in the seminal receptacle but not in spermathecae. Sperm stored in seminal receptacle are designated as the more immediate fertilization set^36^ and therefore, our observation might reflect the fight between control or exposed sperm for fertilization. In addition, S_2_ was suggested to be a highly significant predictor of proportion of offspring sired by the second male (P_2_) for the seminal receptacle^21^. Our observations in offensive sperm competition assays at the sub-organismal as well as organismal level support the above notion that S_2_ can be significant predictor of P_2_. In addition, our findings in defensive sperm competition assays suggest that S_1_ can also be a predictor of P_1_. Together, our findings indicate that exposed males performed worse in sperm competition, both as 1^st^ and 2^nd^ male competitors. This may be a consequence of the effect of endosulfan on the sperm competitive ability. Alternatively, poor performance of exposed males may also be due to the preferential sperm use by the mated females. However, this is not a consequence of receipt of endosulfan along with sperm by the mated females as we did not detect the isomers of endosulfan in mated females but detected the same in exposed males.

Sperm competition is positively associated with larger testes, increased sperm production, improved sperm morphology, design and energetics^13^,^37^,^38^,^39^, and discrepancies in their storage and/or usage^40^. In Drosophila, several proteins from the male accessory glands (Acps) participate in sperm competition^41^,^42^. Further, males are competent of modifying their ejaculates in response to social and environmental cues^43^. In addition, exposure to endosulfan is known to affect processes pertinent to spermatogenesis in rats^44^. Endosulfan causes generation of reactive oxygen species (ROS) and induces oxidative stress in exposed organisms^24^. Sperm DNA is vulnerable to oxidative damage resulting in reduced viability^45^. In these contexts, (1) differential storage of sperm by the females mated to control and/or exposed males, (2) altered seminal proteins and/or sperm viability due to endosulfan exposure in the male may account for the observed poor competitive outcome of sperm from males exposed to endosulfan. However, similar sperm storage levels in the mates of control/exposed males suggest that exposure to 2 μg/ml of endosulfan does not hamper sperm storage and that the observed sperm competition phenotype is not a consequence of differential storage of sperm in mated females. In addition, our semi-quantitative as well as quantitative transcript analyses suggest that the observed findings in sperm competition assays might not be a consequence of chemical mediated modification of seminal proteins and/or reproductive proteins. The lack of significant differences in the ejaculate mediated post-mating processes (such as egg production, progeny production, and sperm storage) between females mated to exposed males, and their controls in the present study do not concur with the possibility of reduced sperm viability and/or decreased transfer of ejaculate by the exposed males to their mates influencing sperm competition. At this juncture, it is important to note that exposure to xenobiotics has been associated with alterations in sperm chromatin structure^46^ although the underlying mechanisms are unclear. Therefore, the observed poor performance of exposed males may be the consequence of endosulfan mediated effects on the sperm DNA. Alternatively, harmful effect of endosulfan on the post-copulatory reproductive success might be mediated by cryptic female choice^47^ that enables females to manipulate the sperm from different males. Females' choice against xenobiotic exposed sperm might be a way to safeguard their reproductive investment as well as fitness. Future studies on cryptic female choice under exposure conditions and on the effect of endosulfan on the sperm chromatin architecture and associated epigenetic modifications^48^ would help to resolve the intricacies underlying the xenobiotic effects on the competitive ability of sperm. Nevertheless, present study does emphasize and point towards the detrimental effect of a xenobiotic on sperm competition, a naturally prevalent and selective phenomenon, at a concentration with no adverse effects on the conventionally assayed toxicological parameters, including progeny production and sperm counts.

The adverse influence of xenobiotic on sperm competition as reflected in the present study has ecological as well as toxicological implications. The observed retention as well as use of control sperm over exposed sperm in females although reflects nature's efforts to nullify the chemical exposure to protect the female reproductive interests, it has compromised the reproductive fitness of those males exposed to xenobiotics (endosulfan in this case). Such a non-alignment in reproductive interests between sexes can lead to sexual conflict^49^, which can have a huge impact on the mean fitness of the population^50^,^51^ and by extension, the population demography^11^,^52^. From the male reproductive toxicity perspective, sperm competition is quite vulnerable to endosulfan exposure and hence males appear to be at odds in this era of industrialization in species with polyandry. Further, assaying the effects of xenobiotics on competitive measures of reproduction such as sperm competition along with non-competitive traditional reproductive toxicity measures would not only mimic the prevailing scenario in nature but also help to understand the consequences of low-level exposures to xenobiotics in the environment.

To conclude, we have shown here that sperm from males exposed to endosulfan fare poorly in the context of sperm competition even at a low level of exposure concentration as well as duration where other reproductive parameters tested are normal. Further, sperm competition is quite vulnerable to endosulfan exposure, and that the exposure may hamper the reproductive output of males in species with polyandry, with the odds stacked against their sperm. Our study highlights the need for the addition of competitive measures of reproductive performance for the comprehensive understanding of toxicology of male reproduction. In addition, our study reflects the capability as well as utility of Drosophila as a model for the assessment of male reproductive toxicity potential of xenobiotics.

## Methods

### Stocks

Drosophila strains carrying sperm labeled with enhanced green fluorescent protein (EGFP) [*w*^1118^; p{ProtamineB-EGFP, *w*^+^} 75A(3)] or red fluorescent protein (dsRed) [*w*^1118^; P{*w*^+^, ProtamineB-dsRed}50A(III)] labeled^21^ were generously provided by Prof. John Belote, Syracuse University, USA. The white eye strain (*w*^1118^) and the wild type strain (Oregon R) of *Drosophila melanogaster* were from Bloomington Stock Center, USA. All flies were reared on standard Drosophila Corn-sucrose, yeast medium, at 22 ± 2°C, and a 12:12 hour light/dark cycle.

### Exposure of *Drosophila melanogaster* males to endosulfan

Adult males within 24 hours of their eclosion were exposed to endosulfan in accordance to the protocol of National Toxicology Programme of US Department of Health and Human services^53^. Briefly, unmated GFP or dsRed males were placed in a vial containing a tissue wick soaked in 2 ml of 5% sucrose containing endosulfan at concentrations ranging from 0.02 to 2 μg/ml, for 72 hours at 22 ± 2°C. Endosulfan was dissolved in DMSO and hence unmated males exposed to 0.04% DMSO in 2 ml of 5% sucrose formed the vehicular controls. For fertility and transcript analysis, we included males fed on 5% sucrose alone as additional controls. Each group consisted of a minimum of 2–3 replicates and each replicate contained 20 males.

### Defensive sperm competition assay

The sperm defense assay was carried out as in Manier et al^21^. Briefly, 3–5 days old *w*^1118^ virgin females were individually mated to GFP males exposed to endosulfan at a concentration of 2 μg/ml. Females mated to males fed on 0.04% DMSO in 5% sucrose formed the control group. The mating pairs were observed and pairs that copulated for 15–20 mins were considered successful while those copulations that lasted for <15 mins were considered unsuccessful and the same were discarded. Males were discarded immediately after the mating was completed. The mated females were individually transferred to vials containing fresh food medium and were retained for three days at 22 ± 2°C. Since 3 days post- mating is generally considered to be the typical remating latency period for *D. melanogaster*^21^, these once mated females were given the opportunity to remate to 3 day old control dsRed males at 3 days ASM (After the Start of Mating). The mating pairs were observed for successful mating, as above. Subsequently, at 2 h ASSM (After the Start of Second Mating), we counted the number of GFP and dsRed sperm in the sperm storage organs (namely seminal receptacle and paired spermathecae) of remated females to calculate the proportional representation of first male sperm (S_1_) in each region of the reproductive tract, by comparing the number of EGFP sperm to the total of EGFP + dsRed sperm in storage. We also determined ratio of EGFP to dsRed sperm in the storage organs. The differences, if any, were statistically analyzed using Mann-Whitney U test.

To determine the proportion of the total progeny sired by the first male (P_1_, control or exposed), the same set of crosses with similar treatment schedules were set up as stated above, except that *w*^1118^ males (3 days old) were used in place of dsRed males as the second male. This was essential to discriminate between the progeny of first and the second male. The remated females were transferred to fresh food vials twice, over the span of ten days. The red eyed (sired by first male), and the white eyed (sired by second male) progeny were counted. The proportion of the progeny sired by the first male (control/exposed) was determined as the ratio of red eyed progeny out of the total progeny per female over 10 days ASSM. Each group consisted of 25–30 replicates. These assays were carried out through single blind coding: identity of crosses/batches was coded by one personnel and subsequently these were decoded only after the counting of progeny was completed by the second personnel and finally data were assigned to the respective groups. The differences between control and exposed groups were statistically analyzed through Mann-Whitney U test.

### Offensive sperm competition assay

The ability of the sperm to displace the rival sperm was analyzed through offensive sperm competition assays (exposed male is second to mate). These assays were done, as aforesaid, except that *w*^1118^ virgin females (3–5 days old) had control dsRed male as first mate and EGFP male with or without exposure to endosulfan as second male. Subsequently, the proportions of second male's sperm in storage (S_2_) and the ratio of EGFP to dsRed in the sperm storage organs of remated females were determined as above. The statistical analysis was performed using Mann-Whitney U test.

For the determination of the proportion of the total progeny sired by the second male (P_2,_ control/exposed), the *w*^1118^ virgin females (3–5 days old) were mated to *w*^1118^ males as their first mates, and GFP males (control/exposed), as the rival second mates. The remated females were transferred to fresh food vials twice, over the span of ten days. The white eyed (sired by first male) and the red eyed (sired by second male) progeny were counted. The proportion of the progeny sired by the second male (control/exposed) was calculated as above. Each group consisted of 25–30 replicates. The differences were statistically analyzed using Mann-Whitney U test.

### Sperm Counts

Sperm counts were carried out as in Mueller et al^54^, except that sperm were counted using fluorescent label on the sperm heads as a marker. The number of sperms stored both in seminal receptacle and paired spermathecae, at 2 h ASM (after the start of mating) or 2 h ASSM were counted twice, with a repeatability index of 91–95%, under fluorescent microscope (at total magnification of 600X with GFP and PI filters, Leica DMLB, Germany) and sample identity was coded to avoid bias. The differences in the sperm counts, if any, between control and exposed mates, were analyzed statistically using Mann-Whitney U test.

### Analysis of eggs laid and progeny produced by females mated to endosulfan exposed males

The assays for determination of the reproductive performance of flies were carried out as in Ravi Ram et al^26^ with slight modification. Briefly, males from control, vehicular control and exposed batches (0.02, 0.2 and 2 μg/ml endosulfan) were individually mated to 3–5 days old wild type (Oregon-R) virgin females in pairs. Mating was observed and pairs that mated for unusually shorter duration (<15 min) were removed from the analysis. Following mating, males were discarded and females were allowed to lay eggs. With intermediate transfer to individual fresh food vials every three days assays were carried out for 10 days. The number of eggs laid by the mated females were counted on daily basis post transfer of the female to fresh food vial, and the number of progeny eclosed out of the laid eggs were determined by manual counting of the adult flies. These assays were repeated twice with 15–20 replicates in each group. The differences, if any, were analyzed through One-way ANOVA.

### Analysis of transcripts of genes encoding reproductive proteins

To determine the effect of endosulfan on genes encoding seminal proteins, total RNA was isolated from males from control, DMSO and exposed groups, independently, by TRIzol extraction according to the manufacturer's instructions (Life Technologies, USA). cDNA was synthesized using the first strand synthesis system for RT-PCR (Fermentas, USA). Transcript levels of 11 reproductive tract genes (CG8194, CG17673, CG8137, PEB-me, CG11664, CG17575, GLD, CG15116, CG9847, CG4760, CG7404) were measured semi-quantitatively through PCR amplification by using gene-specific primers^55^ (amplification cycles-25; other conditions applied were same as in^55^). The amplified products were resolved on 1.5% agarose gels and the profiles were documented using densitometer (Bio-Rad, USA). RPL32 (CG7939), a constitutive gene, was used as an internal control for the quality and quantity of template used for amplification. To confirm the data obtained from semi-quantitiatve transcript analysis, we analyzed the transcript levels of GLD, and CG17673 as described in ref 17 (please see supplementary material for detailed methodology), through quantitative real time PCR (qPCR). Further, transcript levels of few additional genes, implicated in sperm storage and/or sperm competition (Acp36DE^31^, Acp29AB^32^, CG1262^54^, CG9997, and CG1652^29^) were also quantified through qPCR (please see Table S1 for the details of the sequences of the Real-Time primers used). The experiments were performed in triplicate with two technical replicates per biological replicate for every group (control, DMSO, and 2 μg/ml endosulfan exposed). Differences in the fold change of transcript levels between groups were analyzed statistically by employing One- way ANOVA.

### Gas chromatography coupled with mass-spectrometry based estimation of endosulfan within the fly

Both control and exposed flies, ten per batch, in three replicates, were washed with hexane to remove external contamination of endosulfan and were subsequently homogenized in 1 ml acetone and the samples were ultrasonicated for 10 mins. Subsequently, endosulfan analytes were extracted into trichloroethylene and were subjected to ultracentrifugation at 5000 rpm for 5 min. The sedimented phase at the bottom was collected and 1 μl each was injected into Trace GC ultra gas chromatograph connected to a Quantum XLS mass spectrometer (Thermo Scientific, FL, USA) equipped with TG-5MS capillary column to measure the amount of endosulfan within control/exposed flies and their mates. Helium was used as a carrier gas and the oven temperature programming was as follows: the initial oven temperature was 100°C for 1.0 min, and then was increased to 260°C at a rate of 3°C/min and held for 5.0 min. The ion source and transfer line temperature were 220°C and 290°C, respectively.

### Confocal microscopy

To visualize the fluorescent labeled sperm, reproductive tracts of mated females were dissected at 2 h ASSM in physiological saline and were mounted on a slide. Subsequently, the dsRed (red) and EGFP (green) sperm were observed, and images were captured using confocal microscope (Leica, Germany). A minimum of 10 reproductive tracts were observed for each group.

## Author Contributions

S.M. and K.R.R. designed the experiments. S.M., A.K., C.R., V.S. and M.K.R.M. carried out experiments. S.M., A.K., C.R., M.K.R.M. and K.R.R. analyzed the results. All authors wrote and reviewed the manuscript.

## Supplementary Material

Supplementary Information Supplementary Information
