# Supplementary material for: Exposure to endosulfan influences sperm competition in Drosophila melanogaster
Source: Sci Rep. 2014 Dec 11;4:7433. doi: 10.1038/srep07433 (PMC4262826; doi:10.1038/srep07433)
Supplement: Supplementary Information [file srep07433-s1.pdf]

Exposure to endosulfan influences sperm competition in *Drosophila melanogaster*

Snigdha Misra[1,3], Ajay Kumar[1,3], CH Ratnasekhar [2,3], Vandana Sharma[1,3], Mohan Krishna Reddy Mudiam[2,3], Kristipati Ravi Ram[1,3]*

[1]Embryotoxicology, [2]Analytical Chemistry, CSIR-Indian Institute of Toxicology Research, M.G. Marg, Lucknow, 226024, Uttar Pradesh, India. [3]Academy of Scientific and Innovative Research (AcSIR), CSIR-IITR campus, Lucknow.

Consists of supplementary figures S1-S2, Table S1 and methodological details

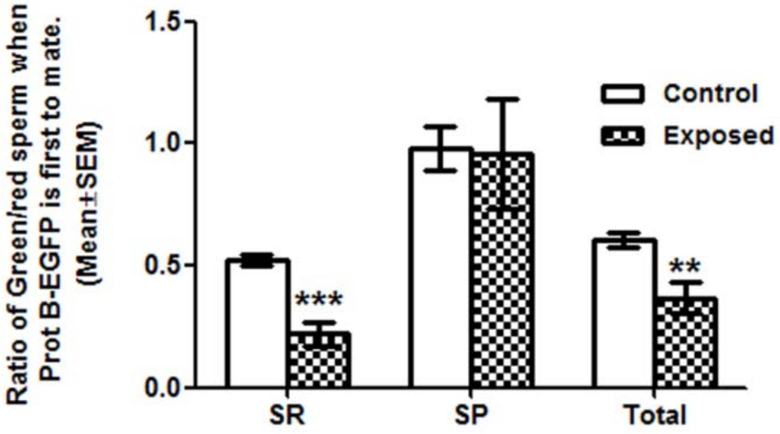

Fig. S1. The ratio (R1) of EGFP sperm (first male) to dsRed sperm (second male) in the sperm defense assay. We observed a significant reduction in the ratio of EGFP to dsRed sperm in the Seminal Receptacle (SR) of female, mated to exposed Prot B-EGFP, as their first mates, compared to that in females having Prot B-EGFP males as their first mate (***$p<0.001$; N=15-20), Relatively equal ratios ($p>0.05$; N=15-20) of EGFP to dsRed sperm were evident in the spermathecae (SP) of females first mated to control or exposed Prot B-EGFP, and subsequently to control Prot B-ds red males. However, we observed significantly reduced ratio of EGFP to dsRed sperm among the total sperm (**$p<0.01$) in storage of females (in both the sperm storage organs SR and SP) with the same mating order as above, when compared to their controls.

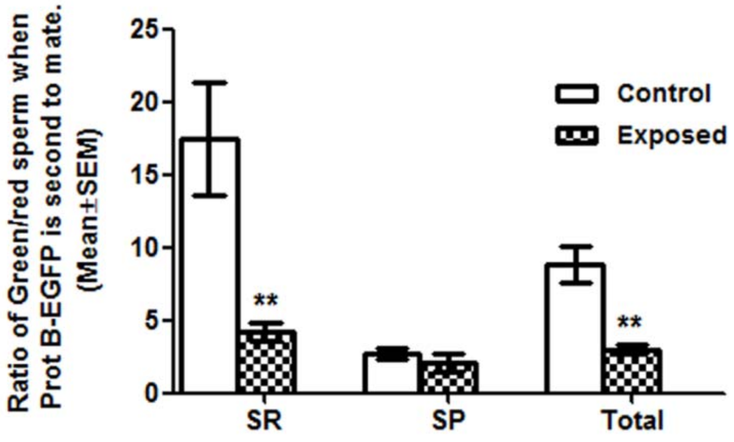

Fig. S2. The ratio (R2) of EGFP sperm (second male) to dsRed sperm (first male) in the sperm offense assay. A significant reduction in the ratio of EGFP to dsRed sperm is evident in the SR (***$p<0.001$; N=10-15) but not in SP ($p>0.05$; N=10-15) of females, mated to exposed Prot B-EGFP, as their first mates, compared to that in females having Prot B-EGFP males as their first mate. In addition, we observed significantly reduced ratio of EGFP to dsRed sperm when total sperm (**$p<0.01$) in storage of females (based on sperm in both the sperm storage organs SR and SP) mated to exposed males, in comparison to their controls.

Table S1. Primers used for Real time PCR

Act-5c	Forward CGATTTGACCGACTACCTGATG<br>Reverse GCACAGCTTCTCCTTGATGT
CG1262	Forward GTCCTGTAGCATGTCCTGAAA<br>Reverse GCCGGAATCCTCTCATTGATAA
Acp36DE	Forward CCCGAGTCTTCACAAGTGATT<br>Reverse GTACGGCCCATGTATTCCTATC
Acp29AB	Forward AGTAGCTTGCTGGAGTTTAAGG<br>Reverse GTTAGACGCTTTGATGTTGGATG
CG9997	Forward TCCCATGCTGGTTGCTATTC<br>Reverse GCTCGATGGGCTCGTATATTT
CG1652	Forward GATCAACGGCAAGTGCTACTA<br>Reverse GTCGAAGTCCCTTTGGTTACTC
GLD	Forward CCCACCTCTATCCTACGCTATT<br>Reverse CCGTCATCTGGGCAATCATAAA
CG17673	Forward TTGGTTCTCGTTTGCGTACT<br>Reverse GCACCACTTATCACGAGGATT

Supplementary methods

Conditions applied for Real time PCR

1 cycle of pre incubation (50ºC for 2 min and 95ºC for 10 min) followed by 40 amplification cycles (95ºC for 15 seconds, and 60ºC for 1 min) and melting curve detection (95ºC for 5 sec, 65ºC for 1 min). Experiments were preformed in duplicate for each sample for seven genes (please see Table S1 for primer sequences) with three biological replicates. The gene

expression data were analyzed using the comparative $2^{-\Delta\Delta}$CT [1]considering Act-5c as the internal control. All results are given as Mean±Standard Error of the mean (Mean±SE).

1. Schmittgen, T. D. & Livak, K. J. Analyzing real-time PCR data by the comparative C(T) method. *Nat. Protoc.* **3**, 1101-8 (2008).
